# Association of Blood Glucose Level and Glycemic Variability With Mortality in Sepsis Patients During ICU Hospitalization

**DOI:** 10.3389/fpubh.2022.857368

**Published:** 2022-04-29

**Authors:** Zongqing Lu, Gan Tao, Xiaoyu Sun, Yijun Zhang, Mengke Jiang, Yu Liu, Meng Ling, Jin Zhang, Wenyan Xiao, Tianfeng Hua, Huaqing Zhu, Min Yang

**Affiliations:** ^1^The Second Department of Intensive Care Unit, The Second Affiliated Hospital of Anhui Medical University, Hefei, China; ^2^The Laboratory of Cardiopulmonary Resuscitation and Critical Care Medicine, The Second Affiliated Hospital of Anhui Medical University, Hefei, China; ^3^Key Laboratory of Intelligent Computing and Signal Processing, Anhui University, Ministry of Education, Hefei, China; ^4^Laboratory of Molecular Biology and Department of Biochemistry, Anhui Medical University, Hefei, China

**Keywords:** sepsis, glucose metabolism disorders, mortality, restricted cubic splines regression, glycemic control

## Abstract

**Background:**

There was considerable debate regarding the effect of mean blood glucose (MBG) and glycemic variability (GV) on the mortality of septic patients. This retrospective cohort study aimed to assess the association between MBG and GV with ICU mortality of sepsis patients and to explore the optimal MBG range.

**Methods:**

Sepsis patients were enrolled from the Medical Information Mart for Intensive Care IV database (MIMIC-IV). MBG and glycemic coefficient of variation (Glu_CV_) were, respectively, calculated to represent the overall glycemic status and GV during ICU stay. The associations between MBG, Glu_CV_, and ICU mortality of the septic patients were assessed by using multivariate logistic regression in different subgroups and the severity of sepsis. Restricted cubic splines evaluated the optimal MBG target.

**Results:**

A total of 7,104 adult sepsis patients were included. The multivariate logistic regression results showed that increased MBG and Glu_CV_ were significantly correlated with ICU mortality. The adjusted odds ratios were 1.14 (95% CI 1.09–1.20) and 1.05 (95% CI 1.00–1.12). However, there was no association between hyperglycemia and ICU mortality among diabetes, liver disease, immunosuppression, and hypoglycemia patients. And the impact of high Glu_CV_ on ICU mortality was not observed in those with diabetes, immunosuppression, liver disease, and non-septic shock. The ICU mortality risk of severe hyperglycemia (≧200 mg/dl) and high Glu_CV_ (>31.429%), respectively, elevated 2.30, 3.15, 3.06, and 2.37, 2.79, 3.14-folds in mild (SOFA ≦ 3), middle (SOFA 3–7), and severe group (SOFA ≧ 7). The MBG level was associated with the lowest risk of ICU mortality and hypoglycemia between 120 and 140 mg/dl in the subgroup without diabetes. For the diabetic subset, the incidence of hypoglycemia was significantly reduced when the MBG was 140–190 mg/dl, but a glycemic control target effectively reducing ICU mortality was not observed.

**Conclusion:**

MBG and Glu_CV_ during the ICU stay were associated with all-cause ICU mortality in sepsis patients; however, their harms are not apparent in some particular subgroups. The impact of hyperglycemia and high GV on death increased with the severity of sepsis. The risk of ICU mortality and hypoglycemia in those with no pre-existing diabetes was lower when maintaining the MBG in the range of 120–140 mg/dl.

## Introduction

Sepsis, defined as organ dysfunction caused by a dysregulated host response to infection by the 2021 Surviving Sepsis Campaign (SSC) Guideline, is associated with high mortality and rapidly became a significant global health burden (1, 2). The glycometabolism disorder is highly prevalent in critically ill patients, especially those with sepsis (3). The activation of stress induces this disturbance, typically manifested as hyperglycemia and increased glycemic variability (GV) (4). Specifically, under the attack of infections, the overwhelming release of pro-inflammatory mediums results in excessive hepatic gluconeogenesis and peripheral insulin resistance during sepsis (5). Catecholamines and cortisol, released by the adrenal cortex through the activated hypothalamic-pituitary-adrenocortical axis, also play significant roles (6).

The unified blood glucose (BG) management protocols for sepsis patients has not been established, even though much research has been conducted to clarify the specific mechanisms of the glycometabolism disorder. At present, the controversy on glycemic management in patients with sepsis mainly focuses on two aspects. First, the influence of elevated BG has not been fully elucidated. Previous literature has examined the impact of hyperglycemia on poor prognosis in different critically ill patients, such as those with myocardial infarction (7), acute pancreatitis (8), and stroke (9). However, these connections are not consistent across sepsis patients. Many trials have reported that hyperglycemia is associated with increased short-term mortality of sepsis patients (10–12), but neutral even lower mortality risks have also been found (13–16). These seemingly opposite phenomena suggested complex non-linear relationships between the hyperglycemic effect and the prognosis in sepsis patients (17). Although diabetic condition may be associated with the apparent inconsistencies, it was unreasonable to consider it as a specific interpretation. This was because the influence of hyperglycemia also differs in sepsis patients combined with diabetes (11, 13), and it suggested that other disease states likely also play a role. Second, the interaction between overall BG and GV levels was not clear. Although Magee et al. and Chao et al. have respectively demonstrated that early fluctuation disorder in BG increased 30-day mortality and all-cause hospital mortality in sepsis patients (18, 19), the majority of sepsis patients experienced a relapse of the disease. Thus, the overall GV levels during ICU hospitalization seem more relevant to septic prognosis than early BG fluctuation. Third, the optimal BG target is not yet confirmed. Several multicenter studies have disproved the protective effect of traditional intensive glucose control in sepsis patients, such as VISEP and NICE-SUGAR (20, 21). Furthermore, the 2021 SSC Guidelines recommend initiating insulin therapy when the glucose level ≧180 mg/dl and maintenance ranges from 144 to 180 mg/dl (1). Nevertheless, this recommendation draws on the American Diabetes Association Standards of Medical Care in Diabetes Guideline, specific to the entire critically ill population (22). Few studies have focused on the optimal target of BG control, and thus further investigations are necessary considering the heterogeneity of septic patients.

Therefore, we performed a retrospective cohort study based on an extensive, publicly available database called Medical Information Mart for Intensive Care IV (MIMIC-IV). Our primary aim was to examine the association of overall BG and GV levels during ICU admission with all-cause ICU mortality in sepsis patients. The secondary aim of this study was to investigate the optimal range of BG in patients with sepsis and each subgroup. We hypothesized that the influence of BG and GV in different subgroups of sepsis patients on ICU mortality might differ, and the ideal glucose range might also be different across sepsis subgroups.

## Methods

### Study Population and Data Extraction

The Massachusetts Institute of Technology established the MIMIC-IV (1.0 version) database, which contained the medical records of 382,278 in-patients who received care at the Beth Israel Deaconess Medical Center between 2008 and 2019 (23). The latter is one of the preeminent academic medical and referral centers in the Boston area, in which 77 critical care beds are contained. Users can screen demographic characteristics, vital signs, laboratory test results, imaging examinations of each patient by using a unique code given during admission. Lu has completed the Collaborative Institutional Training Initiative program course (Certification number 36763801). Because the MIMIC-IV database is a publicly available anonymized database, approval from the ethical committee was not necessary.

In the present study, we extracted patients' parameters, including (1) demographic features (age, gender), type of care unit, body mass index (BMI); (2) neutrophil to lymphocyte ratio (NLR), white blood cell count (WBC), Sequential Organ Failure Assessment (SOFA) score, Acute Physiology Score III (APS III), Charlson Comorbidity Index within the first 24 h after ICU admission; (3) anamnesis (diabetes, immunosuppression, myocardial infarct, congestive heart failure, peripheral vascular disease, liver disease, renal disease, cerebrovascular disease, and chronic obstructive pulmonary disease), infection site; (4) mean BG and glucose variability during ICU stay; (5) the use of mechanical ventilation (MV), renal replacement therapy (RRT), norepinephrine and insulin during ICU stay; (6) incidence of septic shock and hypoglycemia during ICU stay; (7) the length of ICU stay, 7-day mortality, 28-day mortality, ICU mortality of all patients. Immunosuppression was defined as having any of the following major immune diseases: lymphoma, acquired immune deficiency syndrome, solid metastatic tumor, malignant tumor, or autoimmune diseases. All related diseases were identified by the International Classification of Diseases, Ninth Revision (ICD-9), combined with Tenth Revision (ICD-10) diagnosis codes when the patient is discharged.

All adult sepsis patients (≧18 years) were screened for analysis. We excluded patients who stayed <48 h in the ICU to avoid inaccurate valuation of the condition of glycemic fluctuations. Furthermore, patients were also excluded if they had missing daily BG records. In this study, the diagnosis of sepsis was based on the criteria of the Third International Consensus Definitions for Sepsis and Septic Shock (Sepsis-3), which define sepsis as SOFA ≧2 and the presence of infection or suspected infection (24). Suspected infection refers to antibiotics administered within 3 days or before 24 h of culture collection. It is difficult to implement the procedure which is strictly based on the Sepsis-3 standard to screen septic shock patients in the MIMIC-IV database, and thus we draw on previous experience in this study (25). Septic shock was defined as sepsis with hypotension, and the hypotension was assumed for sepsis patients when any vasopressor was administered during the ICU stay, including norepinephrine, epinephrine, phenylephrine, vasopressin, and dopamine dobutamine or milrinone. For patients with multiple ICU and hospital admissions, we only included data from the first hospital admission and first ICU stay. The flowchart is shown in Supplementary Figure 1.

### Glucose Measurement and Glycemic Variability Definition

For each included patient, we have calculated the mean BG (MBG) during ICU stay using all biochemical glucose records. MBG were stratified as follows: no hyperglycemia (≦140 mg/dl), mild hyperglycemia (140–200 mg/dl), and severe hyperglycemia (≧200 mg/dl) based on previous work (26). We defined hypoglycemia as at least one glucose record <70 mg/dl during ICU stay. Here, we considered glucose≦140 mg/dl as a reference value to which each category is compared. In this analysis, the overall GV was evaluated by calculating the coefficient of variation (Glu_CV_), which is the ratio of the standard deviation (Glu_CD_) to the glycemic average. Due to the lack of universally accepted clinical criteria for grading the Glu_CV_ status of critically ill patients, we grouped Glu_CV_ into three categories according to the percentiles (low: <25th; mild: 25–75th; high: >75th).

### Restricted Cubic Splines

Linear regression was often used to identify the relationship between independent and dependent variables in clinical trials, but this linear relationship was not always easy to meet and particularly likely to occur when the independent variable was continuous. We usually transformed continuous variables into categorical variables based on some special cutoff points to explore the unknown non-linear relationship. However, this approach may change the shape of the dose-response relationship and induce inevitable information loss. Restricted cubic splines (RCS) analysis as a smoothness function is well-fit to non-linear relationships and retains independent local structure. Recently, RCS was widely used to assess the dose-response relationship between continuous variables and dependent variables (27, 28). RCS can be seen as a piecewise polynomial, it requires a continuous second-order derivative existing in each segmented spot (29). The main operation of RCS is that the setting of the knots count and position is required before its use and it may have an influence on the overall structure. With the reference from the previous study (28), we used RCS with five knots, corresponding to the 5, 35, 50, 65, and 95th percentiles, to explore the relationship between MBG with all-cause ICU mortality in sepsis patients. The reference was set at 140 mg/dl.

### Statistical Analysis

The present retrospective study of the collected observational data set was stratified according to MBG and Glu_CV_. We performed a normality test (*Agostino tests*), followed by a descriptive analysis of the data. Continuous variables were expressed as mean (standard deviation) while non-parametric variables were expressed as the median (interquartile ranges, IQR) and were compared using the *one-way ANOVA test* or *non-parametric Kruskal-Wallis test*. The categorical variables are expressed as a frequency (percentage) and were compared using the *X*^2^ or *rank-sum tests*. The random forests function handled missing values. However, the variable was deleted when >30% of the values were lacking. Outlier expressions were defined as values that are greater than the 99th or lower than the 1st percentile. Variables with outliers were winsorized using the *winsor2* command in STATA software.

Multivariate logistic regression was performed to determine the connection between MBG, Glu_CV_, and ICU mortality of sepsis patients separately. MBG was modeled as both continuous and categorical scale; while the MBG category set cutoffs on 140 and 200 mg/dl, and the Glu_CV_ set on the _first_ and third quartile. The potential confounders were adjusted gradually in three models. Initially, we adjusted for age and gender (Model 1). Subsequently, related comorbidities, such as diabetes and immunosuppression, have been adjusted (Model 2). Finally, we adjusted for NLR- related early disease severity scores (APS III, SOFA, and Charlson Comorbidity Index), MBG/Glu_CV_, occurrence or not of septic shock and hypoglycemia, and related interventions including the use of MV, RRT, and insulin except norepinephrine during ICU stay (Model 3).

In the subgroup analyses, we stratified the study population by age (≧65, <65 years), gender (male, female), diabetes, immunosuppression, liver disease, hypoglycemia, and septic shock. The interaction of the levels of MBG and Glu_CV_ with the above covariates for stratification of ICU mortality was examined by including two-factor interaction terms in the multivariate logistic regression model. Meanwhile, the interactions were visualized by the slopes of the regression lines.

To evaluate the performance of MBG and Glu_CV_ in predicting ICU mortality in sepsis patients, we conducted receiver operating characteristic (ROC) curves. We also conducted the dose-response association using the RCS model with five knots located at the 5, 35, 50, 65, and 95th percentiles of the overall distribution for MBG levels based on the multivariate logistic regression model. The exact number and location of knots from the overall population splines were also applied in the splines for each subgroup to allow direct comparison of the overall and stratified analyses. All statistical analyses were performed using STATA 15.1 (College Station, Texas) and R 3.6.2 (Chicago, Illinois) software. The *p-*values with < 0.05 were taken as statistically significant (two-sided).

## Results

### Characteristics of Included Sepsis Participants

In the MIMIC-IV database, a total of 12,274 patients were diagnosed with sepsis at their first ICU admission according to the definition of sepsis 3.0; ultimately, 7,104 patients were included in the analysis; 2,661 patients lacked the height data. Thus, all pre-defined features were included except the BMI index. During the whole ICU stay, the minimum and maximum values of MBG were 81.33 and 294.78 mg/dl, respectively; in addition, the minimum Glu_CV_ was 4.22 %, and the maximum Glu_CV_ was 84.76%. The incidence of septic shock was 40.36% (2,867/7,104), insulin treatment was 54.43% (3,867/7,104), hypoglycemia was 14.26% (1,013/7,104), diabetes was 31.53% (2,240/7,104), and liver disease was 18.65% (1,325/7,104). Among the included septic patients, 841 (11.84%) died during the ICU stay. The MBG of patients who died was significantly elevated compared with survivors [128 (112–155) vs. 142 (119–173); *p* < 0.001]. The dead group also showed significantly increased Glu_CV_ [21.5 (14.8–30.5) vs. 26.4 (18.4–37.1); *p* < 0.001]. The distribution of MBG, Glu_CV_ within the two cohorts is shown in Supplementary Figure 2.

The clinical characteristics based on MBG and Glu_CV_ categories can be found in Table 1. An upward trend was observed at higher MBG levels for initial NLR value, APS III scoring, and the prevalence of diabetes, myocardial infarct, congestive heart failure, peripheral vascular disease, cerebrovascular disease, and renal disease. Similarly, as MBG levels increased, elevated risk of urinary tract infection and septicemia was also presented; but higher MBG levels were not positively correlated with poor prognosis in sepsis patients. For different Glu_CV_ categories, the initial inflammatory markers (WBC, NLR) were higher in individuals with higher Glu_CV_, and the incidence of diabetes, septic shock, myocardial infarct, congestive heart failure, renal disease, and related treatments (RRT, MV, norepinephrine, and insulin infusion) was also elevated. Unlike the MBG levels, there was a positive association between Glu_CV_ and the risk of poor outcomes.

**Table 1:** Baseline demographic and clinical characteristics by mean glucose level and glycemic variability in patients with sepsis.

**Features**	**Mean blood glucose level during ICU hospitalization**			***p*-value**	**Glycemic variability during ICU hospitalization**			***p*-value**
	**Glucose ** <= **140**	**Glucose 140 to <200**	**Glucose **> = ** 200**		**GlUcv < 15.174**	**GLUcv 15.174 to ** <= ** 31.429**	**GlUcv > 31.429**	
	***N* = 4,407**	***N* = 2,029**	***N* = 668**		***N* = 1,776**	***N* = 3,552**	***N* = 1,776**	
**Demographic features**								
Age (years)	68.0 (54.9, 80.5)	69.8 (58.8, 79.8)	68.8 (59.6, 78.1)	0.001	68.5 (55.5, 80.7)	68.5 (56.4, 80.1)	68.8 (57.4, 79.6)	0.9
Gender				0.289				<0.001
Female	2,010 (45.6%)	920 (45.3%)	283 (42.4%)		720 (40.5%)	1,670 (47.0%)	823 (46.3%)	
Male	2,397 (54.4%)	1,109 (54.7%)	385 (57.6%)		1,056 (59.5%)	1,882 (53.0%)	953 (53.7%)	
**Care unit type**				<0.001				<0.001
Cardiac vascular intensive care unit	568 (12.9%)	171 (8.43%)	13 (1.95%)		267 (15.0%)	339 (9.54%)	146 (8.22%)	
Coronary care unit	267 (6.06%)	184 (9.07%)	72 (10.8%)		114 (6.42%)	254 (7.15%)	155 (8.73%)	
Medical intensive care unit	1,403 (31.8%)	647 (31.9%)	267 (40.0%)		507 (28.5%)	1,180 (33.2%)	630 (35.5%)	
Medical/surgical intensive care unit	964 (21.9%)	411 (20.3%)	166 (24.9%)		364 (20.5%)	765 (21.5%)	412 (23.2%)	
Neuro intermediate	112 (2.54%)	38 (1.87%)	11 (1.65%)		55 (3.10%)	81 (2.28%)	25 (1.41%)	
Neuro stepdown	60 (1.36%)	7 (0.34%)	5 (0.75%)		23 (1.30%)	43 (1.21%)	6 (0.34%)	
Neuro surgical intensive care unit	70 (1.59%)	42 (2.07%)	11 (1.65%)		41 (2.31%)	64 (1.80%)	18 (1.01%)	
Surgical intensive care unit	559 (12.7%)	316 (15.6%)	78 (11.7%)		228 (12.8%)	482 (13.6%)	243 (13.7%)	
Trauma SICU	404 (9.17%)	213 (10.5%)	45 (6.74%)		177 (9.97%)	344 (9.68%)	141 (7.94%)	
**Inflammatory indicators** ^#^								
WBC_max (X 10^3^/uL)	13.6 (9.50, 19.0)	15.1 (10.7, 20.6)	15.0 (10.9, 20.3)	<0.001	13.4 (9.60, 18.3)	14.2 (9.80, 19.6)	15.1 (10.6, 21.1)	<0.001
NLR	8.73 (5.80, 14.4)	9.46 (6.33, 15.9)	9.71 (6.90, 16.4)	<0.001	8.20 (5.55, 12.9)	9.13 (6.13, 15.3)	9.77 (6.61, 16.7)	<0.001
**Severe scoring**								
SOFA	3.00 (2.00, 5.00)	3.00 (2.00, 5.00)	3.00 (2.00, 4.00)	0.231	3.00 (2.00, 4.00)	3.00 (2.00, 5.00)	3.00 (2.00, 5.00)	<0.001
APSIII	54.0 (39.0, 73.0)	61.0 (47.0, 82.0)	65.0 (52.0, 83.0)	<0.001	49.0 (36.0, 63.0)	58.0 (43.0, 77.0)	67.0 (50.0, 87.0)	<0.001
Charlson comorbidity index	6.00 (4.00, 8.00)	6.00 (5.00, 8.00)	7.00 (5.00, 9.00)	<0.001	5.00 (4.00, 7.25)	6.00 (4.00, 8.00)	6.00 (5.00, 8.00)	<0.001
**Comorbidity**								
Diabetes	708 (16.1%)	996 (49.1%)	536 (80.2%)	<0.001	324 (18.2%)	992 (27.9%)	924 (52.0%)	<0.001
Septic shock	1,761 (40.0%)	868 (42.8%)	238 (35.6%)	0.003	624 (35.1%)	1,465 (41.2%)	778 (43.8%)	<0.001
Immunosuppression	839 (19.0%)	373 (18.4%)	124 (18.6%)	0.811	323 (18.2%)	707 (19.9%)	306 (17.2%)	0.046
Myocardial infarct	598 (13.6%)	374 (18.4%)	174 (26.0%)	<0.001	234 (13.2%)	554 (15.6%)	358 (20.2%)	<0.001
Congestive heart failure	1,279 (29.0%)	728 (35.9%)	263 (39.4%)	<0.001	503 (28.3%)	1,139 (32.1%)	628 (35.4%)	<0.001
Peripheral vascular disease	455 (10.3%)	214 (10.5%)	84 (12.6%)	0.211	167 (9.40%)	362 (10.2%)	224 (12.6%)	0.004
Cerebrovascular disease	612 (13.9%)	330 (16.3%)	122 (18.3%)	0.002	299 (16.8%)	517 (14.6%)	248 (14.0%)	0.034
COPD	1,215 (27.6%)	576 (28.4%)	187 (28.0%)	0.79	444 (25.0%)	1,052 (29.6%)	482 (27.1%)	0.001
Renal disease	899 (20.4%)	568 (28.0%)	224 (33.5%)	<0.001	353 (19.9%)	794 (22.4%)	544 (30.6%)	<0.001
Liver disease	808 (18.3%)	398 (19.6%)	119 (17.8%)	0.398	281 (15.8%)	686 (19.3%)	358 (20.2%)	0.001
**Infection site**								
Skin subcutaneous tissue	334 (7.58%)	173 (8.53%)	63 (9.43%)	0.16	137 (7.71%)	286 (8.05%)	147 (8.28%)	0.823
Catheter related	88 (2.00%)	56 (2.76%)	16 (2.40%)	0.154	32 (1.80%)	84 (2.36%)	44 (2.48%)	0.325
Urinary tract	874 (19.8%)	359 (17.7%)	150 (22.5%)	0.016	331 (18.6%)	707 (19.9%)	345 (19.4%)	0.545
Intestinal infection	248 (5.63%)	110 (5.42%)	32 (4.79%)	0.667	75 (4.22%)	214 (6.02%)	101 (5.69%)	0.023
Septicemia	1,584 (35.9%)	762 (37.6%)	286 (42.8%)	0.002	516 (29.1%)	1,370 (38.6%)	746 (42.0%)	<0.001
Pulmonary infection	1,468 (33.3%)	706 (34.8%)	210 (31.4%)	0.239	530 (29.8%)	1,282 (36.1%)	572 (32.2%)	<0.001
**Curing**								
RRT	419 (9.51%)	271 (13.4%)	79 (11.8%)	<0.001	106 (5.97%)	388 (10.9%)	275 (15.5%)	<0.001
MV	2,408 (54.6%)	1,273 (62.7%)	389 (58.2%)	<0.001	814 (45.8%)	2,144 (60.4%)	1,112 (62.6%)	<0.001
MV duration (h)	6.50 (0.00, 45.0)	19.0 (0.00, 80.0)	14.3 (0.00, 61.0)	<0.001	0.00 (0.00, 22.4)	14.0 (0.00, 66.3)	20.0 (0.00, 75.4)	<0.001
Norepinephrine	1,156 (26.2%)	614 (30.3%)	188 (28.1%)	0.003	343 (19.3%)	1,028 (28.9%)	587 (33.1%)	<0.001
Insulin	1,627 (36.9%)	1,597 (78.7%)	643 (96.3%)	<0.001	667 (37.6%)	1,884 (53.0%)	1,316 (74.1%)	<0.001
**Outcomes**								
Length of ICU stay	3.92 (2.76, 6.64)	4.52 (2.91, 8.65)	3.89 (2.74, 6.87)	<0.001	3.28 (2.55, 5.05)	4.48 (2.93, 8.01)	4.37 (2.86, 7.93)	<0.001
Mortality_hospital	666 (15.1%)	481 (23.7%)	152 (22.8%)	<0.001	223 (12.6%)	652 (18.4%)	424 (23.9%)	<0.001
Mortality_ICU	407 (9.24%)	330 (16.3%)	104 (15.6%)	<0.001	124 (6.98%)	416 (11.7%)	301 (16.9%)	<0.001
Mortality_ICU_7day	262 (5.95%)	205 (10.1%)	68 (10.2%)	<0.001	105 (5.91%)	242 (6.81%)	188 (10.6%)	<0.001
Mortality_ICU_28day	607 (13.8%)	434 (21.4%)	142 (21.3%)	<0.001	209 (11.8%)	587 (16.5%)	387 (21.8%)	<0.001
Hypoglycemia	759 (17.2%)	198 (9.76%)	56 (8.38%)	<0.001	43 (2.42%)	460 (13.0%)	510 (28.7%)	<0.001

*Continuous data are presented as the median (interquartile range) and categorical data as n (%)*.

*WBC, white blood cell; NLR, neutrophil to lymphocyte ratio; SOFA, Sequential Organ Failure Assessment; APS III, Acute Physiology Score III; COPD, chronic obstructive pulmonary disease; RRT, renal replacement therapy; MV, mechanical ventilation*.

# *Inflammatory indicators use the maximum value in the first 24 h after ICU admission*.

### Association Between MBG, Glu_CV_, and ICU Mortality

On a continuous scale, the results of multivariable logistic regression showed that every 20 mg/dl or 10% rise in MBG and Glu_CV_ was, respectively, associated with 1.14-fold (95% CI 1.09–1.20) and 1.05-fold (95% CI 1.00–1.12) increase in the risk of ICU mortality (Table 2, Model 3). As described previously, we divided the patients into three tertiles according to their MBG levels. Compared with MBG levels ≦140 mg/dl, septic patients with MBG levels between 140 and 200 mg/dl and ≧200 mg/dl had an increased risk of ICU mortality, the aORs were 1.97 (95% CI 1.61–2.41) and 2.23 (95% CI 1.64–3.03), respectively (Table 2, Model 3). Similarly, the 25 and 75th percentiles of Glu_CV_ were used as the cutoff values to subdivide patients with sepsis into three risk categories. Mortality among patients in the lowest category of Glu_CV_ was 6.98%, increasing to 11.7 and 16.9% in the median and highest category (Table 1). The patients with Glu_CV_ ≧ 31.429% had a 0.36 (95% CI 0.04–0.77) higher risk of ICU mortality than those with Glu_CV_ < 15.174% (Table 2, Model 3).

**Table 2:** Odds ratio for death in ICU according to the mean glucose levels and glycemic variability on a continuous scale or in tertile groups.

ICU mortality		***N***	**Event**	**Crude OR (95% CI)**	***P* ^a^**	**a.OR (95% CI)**	***P* ^b^**	**a.OR (95% CI)**	***P* ^c^**	**a.OR (95% CI)**	***P* ^d^**
						**Model 1**		**Model 2**		**Model 3**	
MBG	Glucose (per 20 mg/dl)	7,104	841	**1.12 (1.08, 1.15)**	**<0.001**	**1.12 (1.08, 1.15)**	**<0.001**	**1.17 (1.13, 1.21)**	**<0.001**	**1.14 (1.09, 1.20)**	**<0.001**
	Glucose (mg/dl)										
	Glucose < =140	4,407	407	Ref		Ref		Ref		Ref	
	Glucose >140 and <200	2,029	330	**1.91 (1.63,2.23)**	**<0.001**	**1.88 (1.61, 2.19)**	**<0.001**	**2.16 (1.82, 2.55)**	**<0.001**	**1.97 (1.61, 2.41)**	**<0.001**
	Glucose >=200	668	104	**1.81 (1.43, 2.28)**	**<0.001**	**1.80 (1.42, 2.16)**	**<0.001**	**2.39 (1.83, 3.10)**	**<0.001**	**2.23 (1.64, 3.03)**	**<0.001**
GLUcv	GLUcv (per 10%)	7,104	841	**1.18 (1.13, 1.23)**	**<0.001**	**1.18 (1.13, 1.23)**	**<0.001**	**1.21 (1.16, 1.26)**	**<0.001**	**1.05 (1.00, 1.12)**	**0.05**
	GLUcv %; (quartile: 25%, 75%)										
	GLUcv <15.174	1,776	124	Ref		Ref		Ref		Ref	
	GLUcv >=15.174 and < = 31.429	3,552	416	**1.77 (1.44, 2.19)**	**<0.001**	**1.76 (1.43, 2.18)**	**<0.001**	**1.76 (1.43, 2.19)**	**<0.001**	1.02 (0.81, 1.29)	0.86
	GLUcv >= 31.429	1,776	301	**2.72 (2.19, 3.40)**	**<0.001**	**2.71 (2.18, 3.39)**	**<0.001**	**2.98 (2.37, 3.76)**	**<0.001**	**1.36 (1.04, 1.77)**	**0.03**

*Model 1: adjusted for age and gender*.

*Model 2: adjusted for age, gender and comorbidity*.

*Model 3: adjusted for age, gender, comorbidity, SOFA, APS III, Charlson Comorbidity Index, MBG/Glu_CV_, septic shock or not, mechanical ventilation, renal replacement therapy, insulin infusion, NLR, and hypoglycemic event*.

*aOR and corresponding 95% CIs are presented in bold when the 95% CI cross 1*.

a *P for crude analysis*.

b *P for analysis based on Model 1*.

c *P for analysis based on Model 2*.

d *P for analysis based on Model 3*.

A subgroup analyses indicated that the effect of hyperglycemia on ICU mortality is more pronounced in non-diabetic, non-immunosuppression, non-liver disease, non-hypoglycemia, and septic shock patients. Interestingly, different levels of hyperglycemia did not seem to have obvious adverse impacts on the risk of ICU mortality in patients with diabetes or liver disease. Additionally, a significant interaction effect was found between diabetes (*p* < 0.001), hypoglycemia (*p* = 0.001), liver disease (*p* = 0.002), and MBG levels (Table 3). Supplementary Figures 3–5 visually depicted these interactions, respectively. We observed that the ICU mortality risk among non-diabetics was consistently higher than among people with diabetes at the same level as hyperglycemia (Supplementary Figure 3). Meanwhile, increased MBG had a weak impact on ICU mortality risk for patients who experienced at least one episode of hypoglycemia (Supplementary Figure 4). One of the possible reasons is that the influence of hypoglycemia may mask the effect of hyperglycemia on death. And this phenomenon also occurred in the liver disease cohort (Supplementary Figure 5). The impact of Glu_CV_ on different subgroups varied greatly. Despite the ICU mortality risk appearing incremental with increasing Glu_CV_, the difference was only significant in non-elderly, males, non-diabetics, non-immunosuppression, non-hypoglycemia, non-liver disease, septic shock, and patients not treated with insulin (Table 4). Furthermore, a significant interaction between age and Glu_CV_ was observed (*p* = 0.02) (Supplementary Figure 6).

**Table 3:** Results of subgroup analyses of mean blood glucose level and ICU mortality according to clinical characteristics.

**Subgroup**	***N***	**Event**	**a.OR (95%CI)**	**Subgroup**	***N***	**Event**	**a.OR (95%CI)**	***P* for interaction**
**Age** **<** **65**				**Age** **> = ** **65**				0.98
Glucose < = 140	1,923	157	1.00 (1.00, 1.00)	Glucose < = 140	2,484	250	1.00 (1.00, 1.00)	
140 < Glucose <200	763	111	1.99 (1.42, 2.81)	140 < Glucose <200	1,266	219	1.98 (1.54, 2.55)	
Glucose >=200	259	33	2.32 (1.35, 3.93)	Glucose >=200	409	71	2.22 (1.52, 3.24)	
**Male**				**Female**				0.88
Glucose < = 140	2,397	221	1.00 (1.00, 1.00)	Glucose < = 140	2,010	186	1.00 (1.00, 1.00)	
140 < Glucose <200	1,109	158	1.83 (1.37, 2.44)	140 < Glucose <200	920	172	2.16 (1.63, 2.86)	
Glucose >=200	385	65	2.77 (1.83, 4.19)	Glucose >=200	283	39	1.67 (1.04, 2.66)	
**Diabetes**				**Non-Diabetes**				<0.001
Glucose < = 140	708	66	1.00 (1.00, 1.00)	Glucose < = 140	3,699	341	1.00 (1.00, 1.00)	
140 < Glucose <200	996	114	1.04 (0.70, 1.55)	140 < Glucose <200	1,033	216	2.44 (1.94, 3.06)	
Glucose >=200	536	66	1.21 (0.77, 1.93)	Glucose >=200	132	38	3.52 (2.18, 5.60)	
**Immunosuppression**				**Non-Immunosuppression**				0.83
Glucose < = 140	839	114	1.00 (1.00, 1.00)	Glucose < = 140	3,568	293	1.00 (1.00, 1.00)	
140 < Glucose <200	373	79	1.73 (1.13, 2.63)	140 < Glucose <200	1,656	251	2.15 (1.71, 2.71)	
Glucose >=200	124	27	1.83 (0.97, 3.42)	Glucose >=200	544	77	2.64 (1.85, 3.76)	
**Liver disease**				**Non-Liver disease**				0.002
Glucose < = 140	808	137	1.00 (1.00, 1.00)	Glucose < = 140	3,599	270	1.00 (1.00, 1.00)	
140 < Glucose <200	398	70	1.15 (0.74, 1.79)	140 < Glucose <200	1,631	260	2.36 (1.88, 2.96)	
Glucose >=200	119	17	1.15 (0.56, 2.32)	Glucose >=200	549	87	2.85 (2.01, 4.01)	
**Hypoglycemia**				**Non-Hypoglycemia**				0.001
Glucose < = 140	759	141	1.00 (1.00, 1.00)	Glucose < = 140	3,648	266	1.00 (1.00, 1.00)	
140 < Glucose <200	198	48	1.33 (0.81, 2.18)	140 < Glucose <200	1,831	282	2.22 (1.78, 2.77)	
Glucose >=200	56	11	0.91 (0.39, 2.04)	Glucose >=200	612	93	2.78 (1.98, 3.88)	
**Septic-shock**				**Non-Septic shock**				0.25
Glucose < = 140	1,761	186	1.00 (1.00, 1.00)	Glucose < = 140	2,646	221	1.00 (1.00, 1.00)	
140 < Glucose <200	868	176	2.31 (1.73, 3.09)	140 < Glucose <200	1,161	154	1.69 (1.27, 2.23)	
Glucose >=200	238	50	2.79 (1.77, 4.37)	Glucose >=200	430	54	1.84 (1.20, 2.79)	

*Adjustment factors are the same as those in Model 3*.

**Table 4:** Results of subgroup analyses of Glu_CV_ level and ICU mortality according to clinical characteristics.

**Subgroup**	***N***	**Event**	**a.OR (95%CI)**	**Subgroup**	***N***	**Event**	**a.OR (95%CI)**	***P* for interaction**
**Age** **<** **65**				**Age> = ** **65**				0.02
Glu_CV_ <15.174	741	34	1.00 (1.00, 1.00)	Glu_CV_ <15.174	1,035	90	1.00 (1.00, 1.00)	
15.174 < = Glu_CV_ < =31.429	1,480	152	1.40 (0.93, 2.16)	15.174 < = Glu_CV_ < =31.429	2,072	264	0.85 (0.64, 1.13)	
Glu_CV_ >31.429	724	115	2.21 (1.39, 3.57)	Glu_CV_ >31.429	1,052	186	1.06 (0.76, 1.47)	
**Male**				**Female**				0.95
Glu_CV_ <15.174	1,056	79	1.00 (1.00, 1.00)	Glu_CV_ <15.174	720	45	1.00 (1.00, 1.00)	
15.174 < = Glu_CV_ < =31.429	1,882	201	0.92 (0.68, 1.26)	15.174 < = Glu_CV_ < =31.429	1,670	215	1.22 (0.85, 1.78)	
Glu_CV_ >31.429	953	164	1.45 (1.02, 2.06)	Glu_CV_ >31.429	823	137	1.35 (0.89, 2.05)	
**Diabetes**				**Non-Diabetes**				0.4
Glu_CV_ <15.174	324	23	1.00 (1.00, 1.00)	Glu_CV_ <15.174	1,452	101	1.00 (1.00, 1.00)	
15.174 < = Glu_CV_ < =31.429	992	103	0.92 (0.55, 1.58)	15.174 < = Glu_CV_ < =31.429	2,560	313	1.03 (0.80, 1.35)	
Glu_CV_ >31.429	924	120	0.78 (0.46, 1.39)	Glu_CV_ >31.429	852	181	1.75 (1.29, 2.39)	
**Immunosuppression**				**Non-Immunosuppression**				0.97
Glu_CV_ <15.174	323	32	1.00 (1.00, 1.00)	Glu_CV_ <15.174	1,453	92	1.00 (1.00, 1.00)	
15.174 < = Glu_CV_ < =31.429	707	119	1.17 (0.74, 1.90)	15.174 < = Glu_CV_ < =31.429	2,845	297	0.99 (0.76, 1.30)	
Glu_CV_ >31.429	306	69	1.23 (0.70, 2.18)	Glu_CV_ >31.429	1470	232	1.42 (1.05, 1.93)	
**Liver disease**				**Non-Liver disease**				0.74
Glu_CV_ <15.174	281	25	1.00 (1.00, 1.00)	Glu_CV_ <15.174	1,495	99	1.00 (1.00, 1.00)	
15.174 < = Glu_CV_ < =31.429	686	118	1.03 (0.60, 1.80)	15.174 < = Glu_CV_ < =31.429	2,866	298	1.02 (0.79, 1.33)	
Glu_CV_ >31.429	358	81	1.12 (0.61, 2.10)	Glu_CV_ >31.429	1,418	220	1.43 (1.06, 1.93)	
**Hypoglycemia**				**Non-Hypoglycemia**				0.66
Glu_CV_ <15.174	43	5	1.00 (1.00, 1.00)	Glu_CV_ <15.174	1,733	119	1.00 (1.00, 1.00)	
15.174 < = Glu_CV_ < =31.429	460	92	1.29 (0.46, 4.33)	15.174 < = Glu_CV_ < =31.429	3,092	324	0.97 (0.77, 1.25)	
Glu_CV_ >31.429	510	103	1.29 (0.45, 4.37)	Glu_CV_ >31.429	1,266	198	1.42 (1.07, 1.88)	
**Septic-shock**				**Non-Septic shock**				0.28
Glu_CV_ <15.174	624	45	1.00 (1.00, 1.00)	Glu_CV_ <15.174	1,152	79	1.00 (1.00, 1.00)	
15.174 < = Glu_CV_ < =31.429	1,465	204	1.22 (0.84, 1.79)	15.174 < = Glu_CV_ < =31.429	2,087	212	0.92 (0.68, 1.25)	
Glu_CV_ >31.429	778	163	1.58 (1.05, 2.41)	Glu_CV_ >31.429	998	138	1.24 (0.87, 1.77)	

*Adjustment factors are the same as those in Model 3*.

In this study, we also subdivided the severity of sepsis according to the initial SOFA score. Those with SOFA scores ≦ 3 (25th) and ≧ 7 (75th) were correspondingly assigned to the mild group and severe group, while the 3–7 were defined as the middle group. The results demonstrated that the impact of MBG on death increased with the severity of sepsis; besides, hyperglycemia was independently associated with increased ICU mortality in each group (Supplementary Figure 7A). In addition, the same trends were also found for the relationship between septic severity and Glu_CV_ (Supplementary Figure 7B).

**Figure 1:**
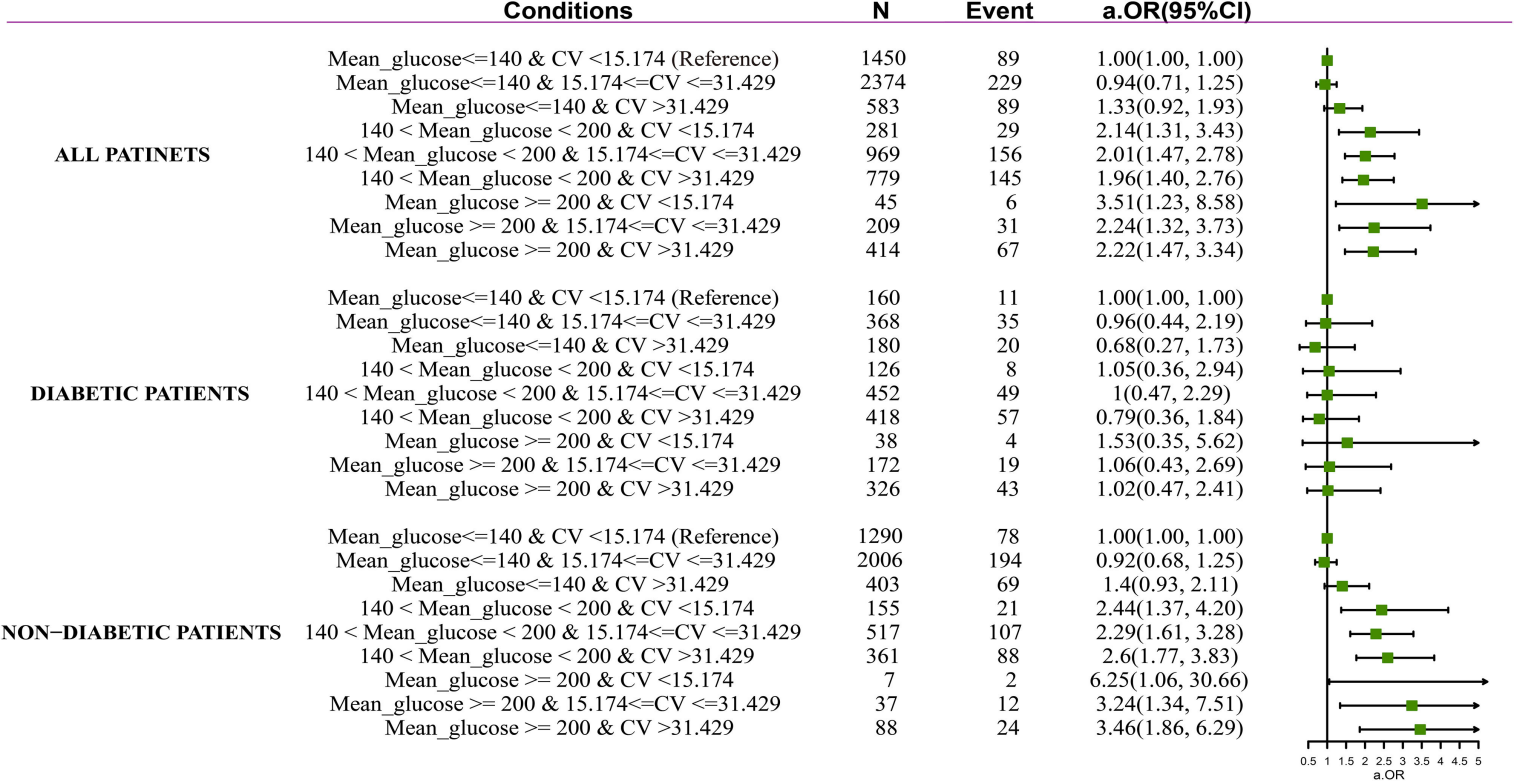
Forest plot depicting ICU mortality risk in septic patients with and without diabetes. Adjustment factors are the same as those in Model 3.

After adjustment for confounders contained in Model 3, among the subjects with non-hyperglycemia, increased Glu_CV_ did not associate with an increased risk of ICU mortality (mild Glu_CV_: aOR 0.94, 95% CI 0.71–1.25; high Glu_CV_: aOR 1.33, 95% CI 0.92–1.93). Among the patients with hyperglycemia, the risk of ICU mortality significantly increases regardless of higher Glu_CV_. Notably, adjusted odds of death were markedly higher in patients with MBG above 200 mg/dl and lower Glu_CV_ values (aOR 3.51, 95% CI 1.23–8.58). In the patients without pre-existing diabetes, mild and severe hyperglycemia were also associated with increased mortality when in combination with various levels of Glu_CV_, that is, mild hyperglycemia plus low Glu_CV_ level (aOR 2.44, 95% CI 1.37–4.20), mild hyperglycemia plus mild Glu_CV_ level (aOR 2.29, 95% CI 1.61–3.28), mild hyperglycemia plus high Glu_CV_ level (OR 2.6, 95% CI 1.77–3.83), high hyperglycemia plus low Glu_CV_ level (aOR 6.25, 95% CI 1.06–30.66), high hyperglycemia plus mild Glu_CV_ level (aOR 3.24, 95% CI 1.34–7.51), and high hyperglycemia plus high Glu_CV_ level (aOR 3.46, 95% CI 1.86–6.29). By contrast, in combination with any Glu_CV_ levels, hyperglycemia was not associated with increased mortality in diabetes patients (Figure 1).

The area under the curve (AUC) of MBG, Glu_CV_, and the combination of two indicators for predicting ICU mortality of all sepsis patients were 0.59, 0.61, and, 0.62, respectively. Three indicators significantly improved risk discrimination in non-diabetics with the AUC increasing from 0.54 to 0.64, 0.55 to 0.64, and 0.56 to 0.66 for the MBG, Glu_CV_, and combination, respectively, compared with those in the people with diabetes. Nevertheless, the overall predictive performance was only moderate (Supplementary Figure 8, Supplementary Table 1).

### Mean Glucose With the Lowest Risk of ICU Mortality

**Figure 2:**
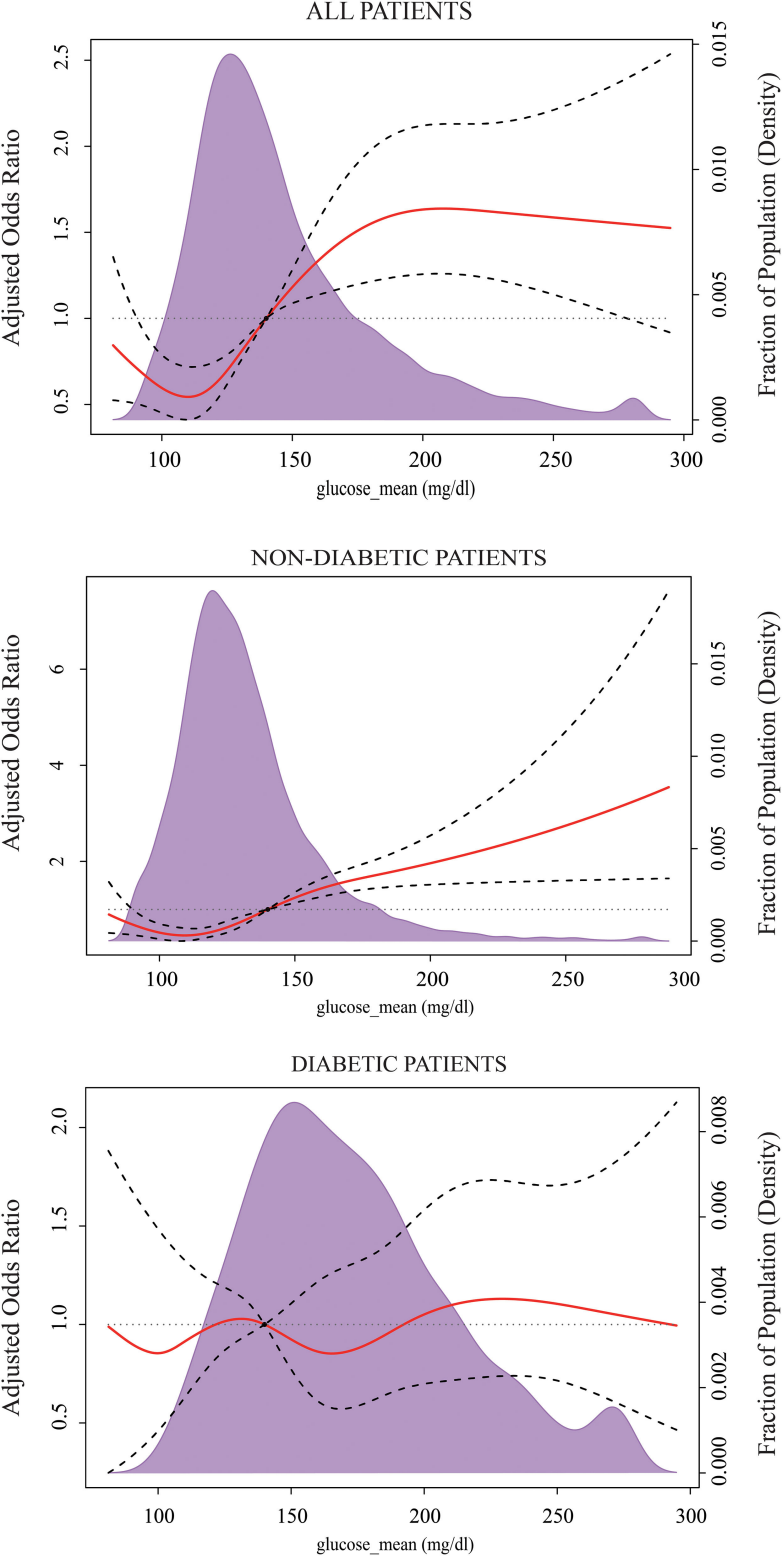
Multivariable-adjusted odds ratios for ICU mortality according to the levels of the mean blood glucose (MBG) on a continuous scale. Solid red lines are multivariable-adjusted odds ratios, with dashed bold lines showing 95% confidence intervals derived from restricted cubic spline regressions with five knots. Reference lines for no association are indicated by the black dashed lines at a hazard ratio of 1.0, and the reference knot set at 140 mg/dl. Purple regions indicate the fraction of the population with different levels of MBG. Adjustment factors are the same as those in Model 3 of Table 2.

The results of RCS after multivariable adjustment presented a non-linear dose-response relationship between the levels of MBG on a continuous scale and the risk of ICU mortality. The concentration of MBG associated with the lowest risk of ICU mortality was ~120 mg/dl in the overall population. The value of aOR has an initial steep increase when MBG is lower than 120 mg/dl or ranges from 120–200 mg/dl, then plateaued. Similarly, the risk reached a minimum when the concentrations of MBG were around 120 mg/dl in non-diabetes patients, and up perpetually with MBG increasing. There was a trend for decreasing the risk of ICU mortality when MBG was between 140 and 190 mg/dl for people with diabetes, but it did not reach statistical significance (Figure 2).

Considering the risk of hypoglycemia, we further compared the incidence of hypoglycemia and death in sepsis patients when containing MBG below 120 mg/dl and in the range of 120–140 mg/dl. The results showed that the hazard of hypoglycemia for patients who maintained MBG lower than 120 mg/dl was significantly greater than the rate for those who maintained MBG between 120 and 140 mg/dl (22.8 vs. 9.35%, *p* < 0.001), while there was no significant difference in ICU mortality (8.57 vs. 10.2%, *p* = 0.08). Within non-diabetics, the risk of hypoglycemia was significantly reduced when MBG was between 120 and 140 mg/dl compared to the MBG level below 120 mg/dl (22.8 vs. 9.35%, *p* < 0.001). And there was no difference in ICU mortality (8.5 vs. 10.3%, *p* = 0.069). For diabetics, patients with 140–190 mg/dl of MBG had a lower hypoglycemic event rate than those who maintained MBG below 140 mg/dl (26 vs. 13%, *p* < 0.001). In addition, no statistical difference was observed between the two divided groups in terms of ICU mortality (9.25 vs. 10.2%, *p* = 0.589).

## Discussion

This study demonstrated the association between MBG, Glu_CV_ during ICU stay, and the increased ICU mortality of septic patients. For the entire cohort, the MBG levels of 140–200 mg/dl, ≧ 200 mg/dl induced a 1.97- and 2.23-fold higher risk of ICU mortality, respectively; and Glu_CV_ of ≦ 31.429% connected with 1.36-fold higher risk. Nevertheless, we found that the effect of MBG and Glu_CV_ on ICU mortality differed among different subgroups. The unfavorable influence of hyperglycemia was more pronounced in non-diabetic, non-immunosuppression, non-liver disease, and non-hypoglycemia patients. And the impact of high Glu_CV_ was more significant in non-elderly, males, non-diabetic, non-immunosuppression, non-liver disease, non-hypoglycemia, and septic shock patients. Furthermore, the impact of hyperglycemia and high Glu_CV_ on death increased with the severity of sepsis. Our results also indicated that the optimal MBG target of sepsis patients without diabetes during ICU stay was 120–140 mg/dl. In diabetic patients, the incidence of hypoglycemia was significantly reduced when the MBG level was set between 140 and 190 mg/dl. A trend of decreased ICU mortality was observed in this BG range, but statistical differences were not reached.

High GV during ICU stay has a solid and consistent relation with adverse prognosis in critically ill patients (30–32). However, there was no consensus regarding the effect of GV on mortality in septic patients. In this study, we calculated the Glu_CV_ using all available biochemical BG records, reflecting the overall intervention status. The results identified that death presented a higher Glu_CV_ than the surviving patients, and a high Glu_CV_ level (>31.43%) was independently associated with an increased risk of ICU mortality among septic patients. In line with our findings, a recent study demonstrated that the rise of the mean amplitude of glycemic excursions and Glu_CV_ within the 1st day of ICU admission was related to increased risk of 30-day mortality in septic patients; in contrast, these relations do not exist in those with diabetes (33). In addition, Ali et al. also reported that GV was an important factor connected with hospital mortality using all biochemical and capillary glucose values for the entire hospitalization (19). Unfortunately, they did not further probe whether GV may vary across different populations.

Of course, the divergent results among such trials may not just depend on the presence of diabetes (30–33). Our study also found that the influence of high Glu_CV_ on ICU mortality was attenuated in the elderly, females, or patients with immunosuppression and hypoglycemia. Although the mechanisms underlying these phenomena are unclear, two reasons could explain this discrepancy. First, a higher incidence of cardiovascular disease, diabetes, and the use of related medications increased as the individuals aged, which changed the natural process of GV and obscured their adverse effects. Second, the risk of hypoglycemia induced by increased GV masked the association between GV and mortality of septic patients. Septic patients are especially vulnerable to hypoglycemia, and the occurrence risk is proportional to the viscera injury severity (34). Previous trials have proved the interaction between hypoglycemia and GV in intensive and non-intensive patients (35–37). The present study similarly showed that the probability of hypoglycemic occurrence rises with increases in Glu_CV_.

The debate surrounding the effect of hyperglycemia on septic patients has been ongoing for more than 10 years. In some studies, hyperglycemia has been argued as an adaptive response under a stress state and plays a protective role in reducing the mortality of septic patients (15, 16). Due to the small number of samples included in these two trials, the stability of this conclusion may be questioned. In contrast, a large multicenter cohort study that contained 7,754 emergency department patients with sepsis demonstrated that high initial BG (>200 mg/dl) was significantly related to increased mortality in non-diabetic patients, but not in those with diabetes (13). In addition, Zohar et al. reported that BG over 200 mg/dl at admission resulted in a 1.48-fold increase in in-hospital mortality, 1.8-fold increase in 30-day mortality, and 1.68-fold increase in 90-day mortality of septic patients (10). However, they claimed that the harm of hyperglycemia was more robust in diabetic patients than in those without diabetes. Although diabetic patients have a greater chance of suffering chronic hyperglycemia, most published papers support that increased BG may not be harmful in septic patients with diabetes (13, 15, 38). Similarly, in this study, we did not observe any relevance between hyperglycemia and the ICU mortality risk of diabetic patients after adjustment in demographic characteristics, other comorbidities, and illness severity. Furthermore, the interaction test also proved that patients without diabetes had a higher risk of mortality in the same MBG range than diabetic patients. Interestingly, our results found that other comorbidities and pathological states, such as immunosuppression, hypoglycemia, liver disease, and septic shock, maybe also affecting the effect of hyperglycemia on the outcomes in septic patients (Table 2). However, no existing study targeted this particular population.

Different diseases may require a different optimal range of BG levels to achieve a better prognosis, and it will impact subsequent medical strategy and interventions (39, 40). However, in patients with sepsis, a firm consensus on optimal BG level is not available. The latest 2021 SSC Guideline recommended that BG should be kept in the range of 144–180 mg/dl for sepsis patients (1), and this recommendation was based on the results of a multicenter RCT (NICE-SUGAR) (21). The NICE-SUGAR study randomized 6,104 critically ill patients to either an intensive glycemic control group with BG of 81–108 mg/dl or a conventional glycemic control group in which insulin was administered if the BG level exceeded 180 mg/dl, and then maintained BG in the range of 144–180 mg/dl. The results presented that the patients in the intensive glycemic control group had lower 90-day mortality (27.5 vs. 24.9%, *P* = 0.02). Nevertheless, it is important to note that there was no statistically significant difference in the all-cause mortality between the two groups in the severe sepsis subgroup (OR 1.13, 95% CI 0.89–1.44). Furthermore, the definition of sepsis has undergone a dramatic change in the past 10 years. Thus, these differences limited the application of this recommendation in clinical practice.

The management protocol of BG in septic patients was usually developed according to the local conditions and experiences of the physicians. The optimum glycemic management needs to consider both the survival benefit and the risk of hypoglycemia. This study found that overall, the patients achieved relatively low mortality and hypoglycemic risk when keeping MBG in the range of 120–140 mg/dl; this range was equally applied to those without diabetes. For the diabetic subset, this study did not find an effective MBG interval that could significantly decrease ICU mortality. Nevertheless, we suggest that diabetes patients maintain MBG between 140 and 190 mg/dl to avoid hypoglycemia. A few published papers have explored the optimal level of BG control in septic patients. In 2019, Wang et al. found that the MBG at admission between 145 and 155 mg/dl was associated with the lowest hospital mortality both in the sepsis patients with and without diabetes based on a dose-response meta-analysis (17). The discrepancies between the two studies were on account of different BG measurements. Considering the effect of subsequent interventions, the MBG during the ICU stay was usually lower than MBG at admission.

### Strengths and Limitations

The strengths of our study were that it was a large cohort that assessed the relationship between MBG, Glu_CV_, and ICU mortality in sepsis patients and each subgroup. In addition, we used RCS to explore the optimal MBG range of sepsis patients in ICU stay. Although residual confounding cannot be completely removed, detailed adjustment for potential confounders about patients themselves and subsequent therapies limited the degree of confounding as far as possible.

Despite these strengths, this study has several limitations. First, we were unable to quantify the timing of each BG measurement, such as fasting or non-fasting in this real-world observational study. Thus, each BG record in this study should be regarded as a random BG. Second, the recent BG control of included patients cannot be accurately reflected due to the lack of complete HbA1c records in the MIMIC-IV database. However, chronic hyperglycemia is strongly associated with the risk of death in critically ill patients (41). Therefore, there could be bias affecting the influence of hyperglycemia in diabetic patients, especially in those with better glycemic control. Third, numerous medications used in the ICU patients and the routes of nutrition are associated with blood glucose metabolism. Nevertheless, this study aimed to determine whether there was a difference in the association of the overall BG and GluCV levels with the prognosis of sepsis patients in the context of the above measures and the reasons for the discrepancy. Furthermore, these related interventions recorded in the MIMIC-IV database were reasonable and recognized. Fourth, this study was a single-center, retrospective cohort study. Our findings need to be validated by an external population.

### Clinical Implications and Future Perspectives

The most salient finding of this study is the evidence for differences in the effects of BG and GV in various septic subgroups, and the reason for this discrepancy is not simply due to the diabetes states. Age, gender, immunosuppression, liver disease, septic shock, and the hypoglycemic event also play an essential role in associating overall BG and GV with ICU mortality in sepsis patients. The current investigation findings have important implications for the development of a reasonable medical strategy and individualized treatment. On the other hand, our results suggest that the glycemic management of septic patients during the acute phase should be assessed individually rather than a “one size fits all” approach.

Moreover, this study questions the plausibility of the latest published 2021 SSC Guideline, which recommends a glycemic target range of 140–180 mg/dl for septic or septic shock patients (1). Given the risk of mortality and hypoglycemia, the optimal range of BG should be different between diabetic and non-diabetic patients. The occurrence of hyperglycemia (>140 mg/dl) should be avoided as much as possible for those without diabetes. In contrast, for diabetic patients, the BG should be maintained at a relatively high level to reduce the risk of hypoglycemia.

Although the mechanisms behind these phenomena are currently unknown, this research has provided further explorations some enlightenment. The enlightenment were listed below, as presented in 1.,2.,3.,4:

- 1. It is necessary to consider BG and GV levels together when implementing glycemic management in septic patients.
- 2. Future studies should focus on investigating the glycometabolism disorder among specific subgroups rather than all the septic patients.
- 3. The optimal glycemic target range of septic patients and related subsets is still controversial. Hence, further studies are warranted to resolve it.
- 4. Despite some new biomarkers and technologies such as capnography and continuous glucose monitoring systems showing a positive effect on clinical glucose management (42, 43), they did not seem to be widely available in sepsis patients. Further studies and consensus are necessary to standardize blood sample collection frequency and time points during the BG monitoring and management of sepsis patients.

## Conclusion

This study demonstrated that MBG and Glu_cv_ during the ICU stay were associated with all-cause ICU mortality in sepsis patients. However, the harm of hyperglycemia and high GV was not apparent in some particular subgroups, such as those with diabetes, immunosuppression, liver disease, and documented hypoglycemia. Furthermore, the results presented that the impact of hyperglycemia and high GV on death increased with the severity of sepsis based on the initial SOFA scores. We also found that patients with severe hyperglycemia (≥200 mg/dl) and low GV (<15.174%) during ICU hospitalization always had the highest all-cause ICU mortality of any subsets regardless of having diabetes or not, indicating that persistent hyperglycemia states were a significant risk factor for ICU deaths of sepsis patients. Although the AUC of MBG combined with Glu_cv_ was superior to either of them alone for predicting ICU mortality in sepsis patients, the overall predictive performance was moderate. Finally, the results of the RCS analysis showed that the risk of ICU mortality and hypoglycemia of those with no pre-existing diabetes were lower when maintaining the MBG in the range of 120–140 mg/dl, whereas in sepsis patients with diabetes, the incidence of hypoglycemia significantly reduced when the MBG level was set between 140 and 190 mg/dl, but a glycemic control target effectively reducing the ICU mortality was not observed.

## Data Availability Statement

The datasets presented in this study can be found in online repositories. The names of the repository/repositories and accession number(s) can be found in the article/Supplementary Material.

## Ethics Statement

Ethical review and approval was not required for the study on human participants in accordance with the local legislation and institutional requirements. Written informed consent for participation was not required for this study in accordance with the national legislation and the institutional requirements.

## Author Contributions

ZL, MY, and HZ designed the study. XS, MJ, and YZ extracted the data. ZL, YL, ML, and TH conducted data quality management and statistical analysis and drafted the manuscript. JZ and WX participated in the literature search. GT, MY, HZ, and TH critically revised the manuscript. All authors contributed to the article and approved the submitted version.

## Funding

This study was supported by a research grant from the National Natural Science Foundation of China (No. 82072134) and the National Natural Science Foundation Youth Science Foundation (No. 81601661).

## Conflict of Interest

The authors declare that the research was conducted in the absence of any commercial or financial relationships that could be construed as a potential conflict of interest.

## Publisher's Note

All claims expressed in this article are solely those of the authors and do not necessarily represent those of their affiliated organizations, or those of the publisher, the editors and the reviewers. Any product that may be evaluated in this article, or claim that may be made by its manufacturer, is not guaranteed or endorsed by the publisher.

## Supplementary Material

Supplementary Figure 1 The flowchart of this present study.

Supplementary Figure 2 The distribution of mean blood glucose (MBG) **(A,B)**, glycemic coefficient of variation (Glu_CV_) **(A,B)** within survivors and deaths during ICU stay. The orange and green solid lines present the density curve.

Supplementary Figure 3 The interaction between mean blood glucose (MBG) and diabetes. The abscissa and ordinate, respectively, represent the MBG values and predictive risk probability of ICU mortality by multivariable logistic regression analysis. Gray solid dots indicate the distribution of each included patient. Solid blue lines are multivariable regression lines, with gray regions showing 95% confidence intervals. Adjustment factors are the same as those in Model 3 of Table 2.

Supplementary Figure 4 The interaction between mean blood glucose (MBG) and hypoglycemia. The abscissa and ordinate, respectively, represent the MBG values and predictive risk probability of ICU mortality by multivariable logistic regression analysis. Gray solid dots indicate the distribution of each included patient. Solid blue lines are multivariable regression lines, with gray regions showing 95% confidence intervals. Adjustment factors are the same as those in Model 3 of Table 2.

Supplementary Figure 5 The interaction between mean blood glucose (MBG) and liver disease. The abscissa and ordinate respectively represent the MBG values and predictive risk probability of ICU mortality by multivariable logistic regression analysis. Gray solid dots indicate the distribution of each included patient. Solid blue lines are multivariable regression lines, with gray regions showing 95% confidence intervals. Adjustment factors are the same as those in Model 3 of Table 2.

Supplementary Figure 6 The interaction between the glycemic coefficient of variation (Glu_CV_) and age. The abscissa and ordinate, respectively, represent the Glu_CV_ values and predictive risk probability of ICU mortality by multivariable logistic regression analysis. Gray solid dots indicate the distribution of each included patient. Solid blue lines are multivariable regression lines, with gray regions showing 95% confidence intervals. Adjustment factors are the same as those in Model 3 of Table 2.

Supplementary Figure 7 The associations between MBG and Glu_CV_ with the ICU mortality of sepsis patients in different severity degrees according to the initial SOFA score.

Supplementary Figure 8 The ROC curve of the mean blood glucose (MBG), glycemic coefficient of variation (Glu_CV_), and MBG + Glu_CV_ in all septic patients **(A)**, diabetic patients **(B)**, and non-diabetic patients **(C)**.

Supplementary Table 1 Comparison of performance of MBG, Glu_CV_, and MBG combined with Glu_CV_ in predicting the ICU mortality of septic patients.

## Abbreviations

SSC: Surviving Sepsis Campaign
GV: glycemic variability
MBG: mean blood glucose
MIMIC-IV: Medical Information Mart for Intensive Care IV
NLR: neutrophil to lymphocyte ratio
WBC: white blood cell count
SOFA: Sequential Organ Failure Assessment
APS III: Acute Physiology Score III
MV: mechanical ventilation
RRT: renal replacement therapy
ICD-9, International Classification of Diseases: Ninth Revision
ICD-10, International Classification of Diseases: Tenth Revision
RCS: Restricted0cubic0splines
ROC: receiver operating characteristic.
