# Supplementary figures and images for: Association of Blood Glucose Level and Glycemic Variability With Mortality in Sepsis Patients During ICU Hospitalization
Source: Front Public Health. 2022 Apr 29;10:857368. doi: 10.3389/fpubh.2022.857368 (PMC9099235; doi:10.3389/fpubh.2022.857368)

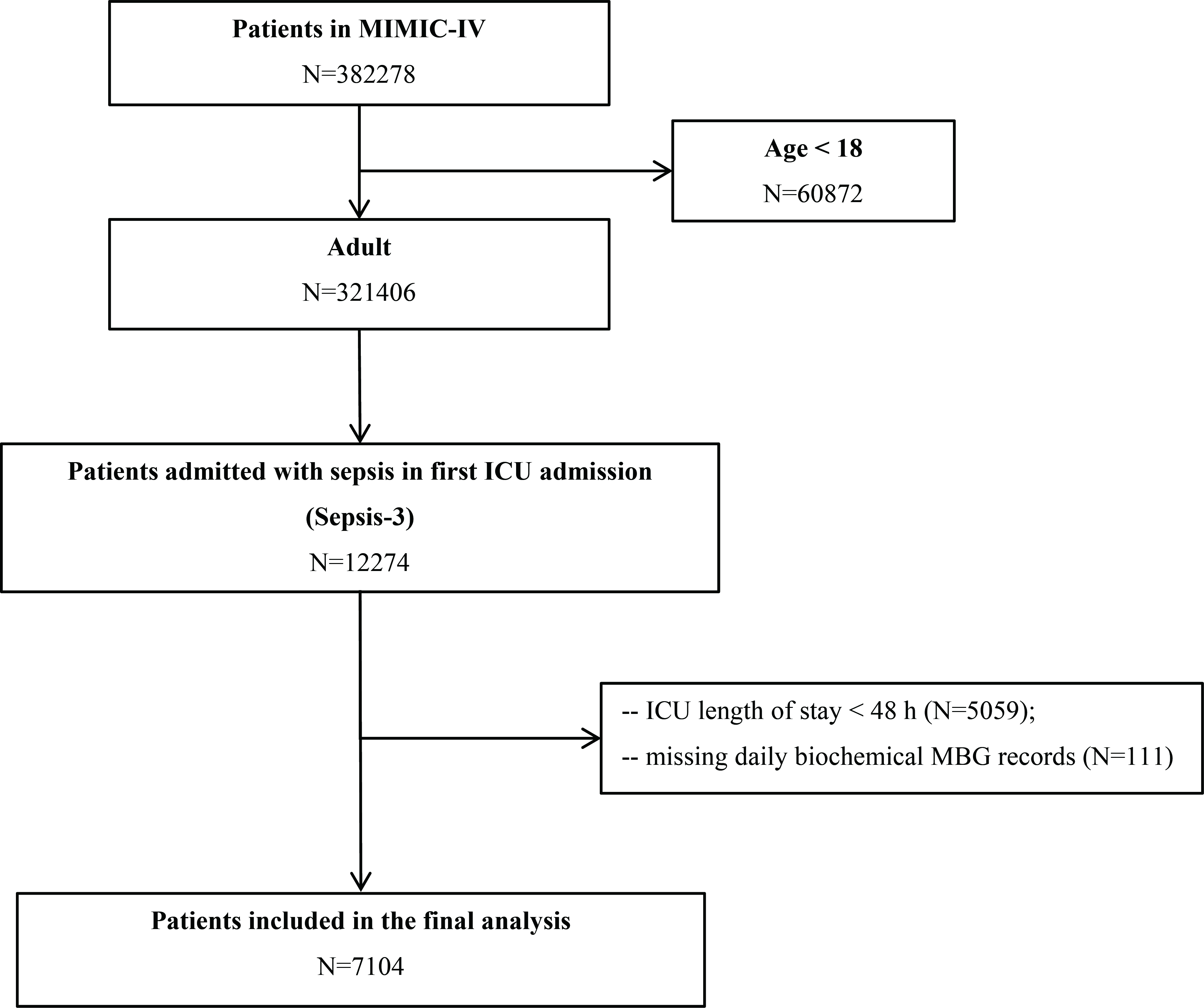

Supplement: Supplementary Figure 1 — The flowchart of this present study. [file Image_1.TIF]

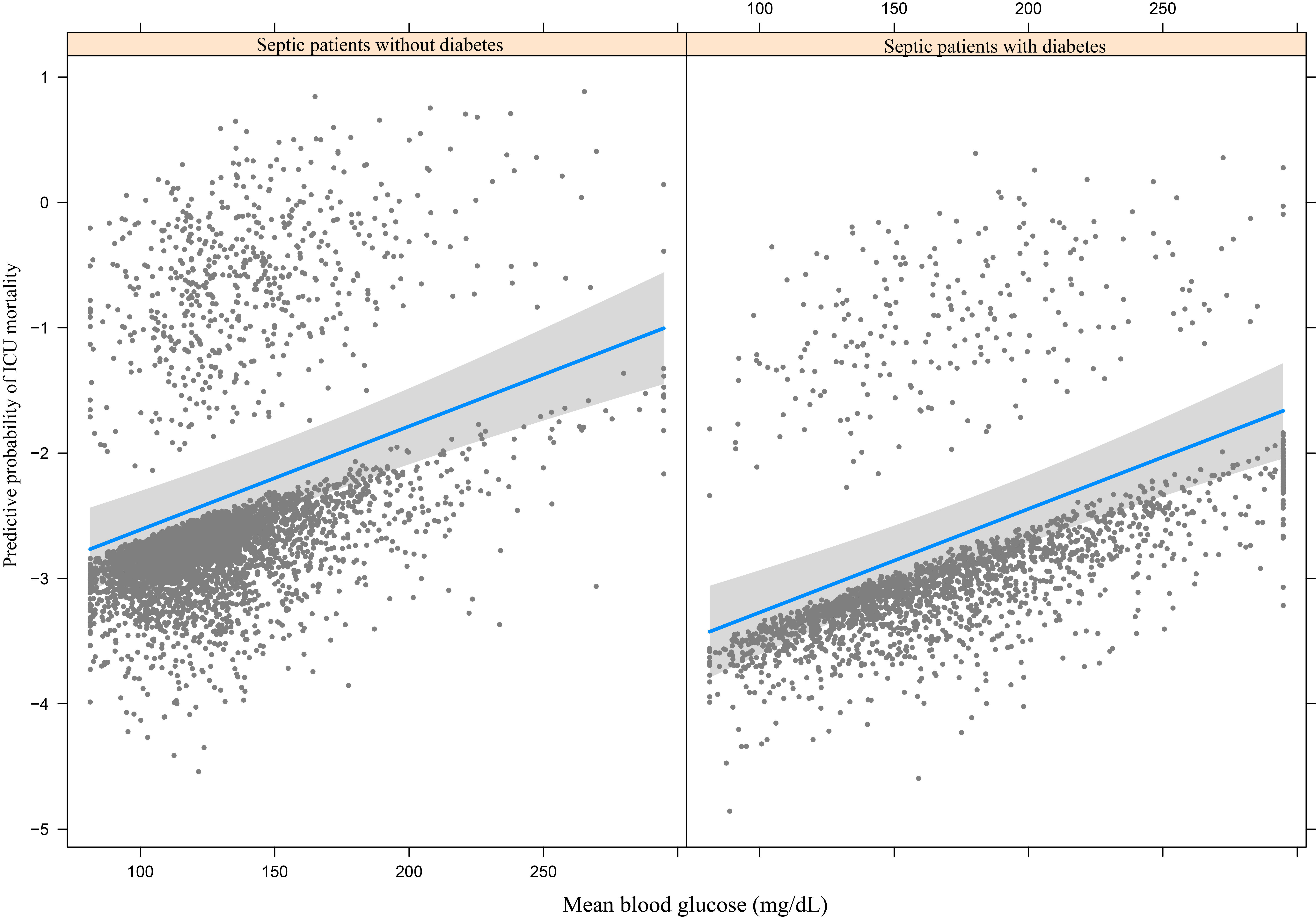

Supplement: Supplementary Figure 3 — The interaction between mean blood glucose (MBG) and diabetes. The abscissa and ordinate, respectively, represent the MBG values and predictive risk probability of ICU mortality by multivariable logistic regression analysis. Gray solid dots indicate the distribution of each included patient. Solid blue lines are multivariable regression lines, with gray regions showing 95% confidence intervals. Adjustment factors are the same as those in Model 3 of Table 2. [file Image_3.TIF]

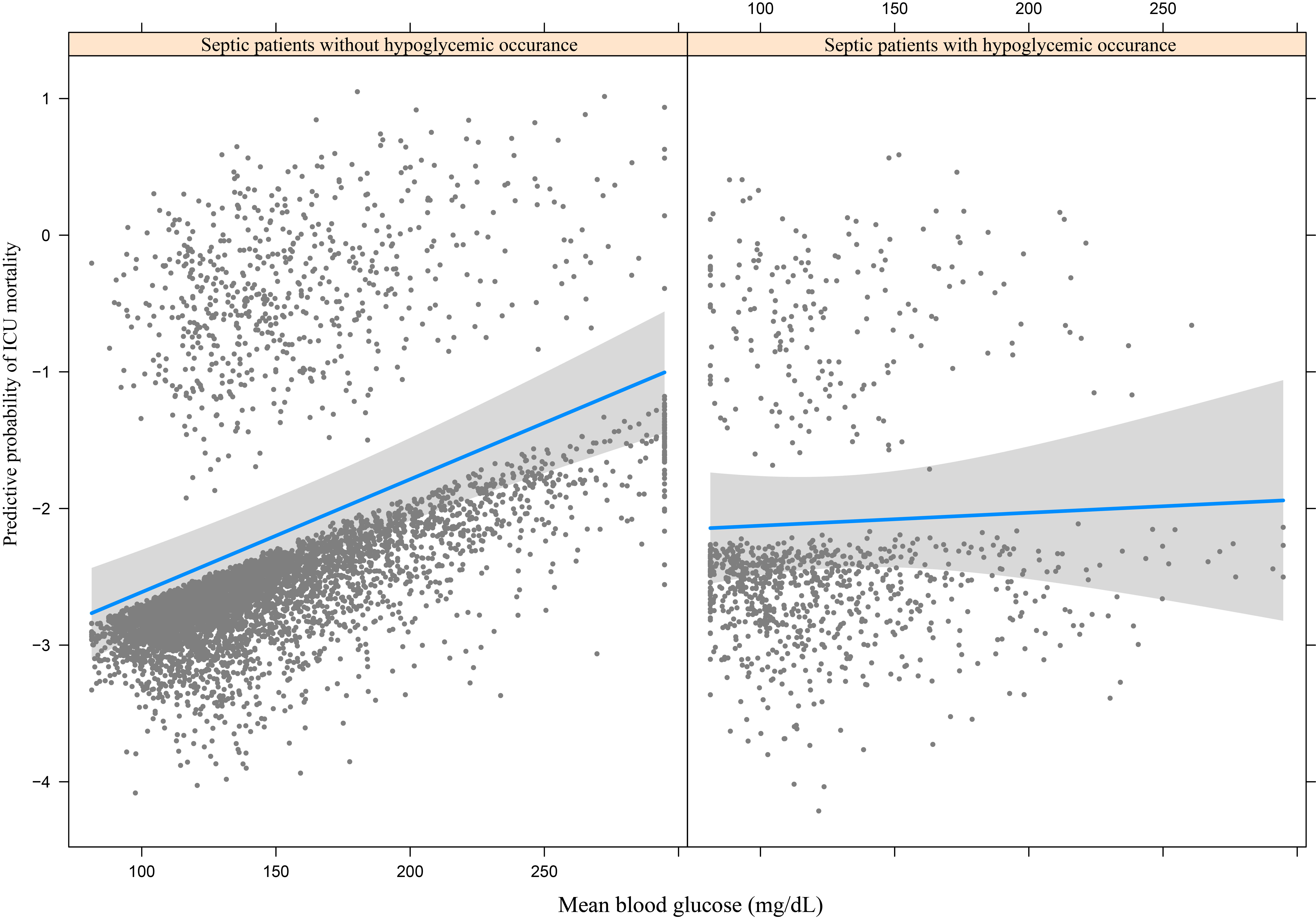

Supplement: Supplementary Figure 4 — The interaction between mean blood glucose (MBG) and hypoglycemia. The abscissa and ordinate, respectively, represent the MBG values and predictive risk probability of ICU mortality by multivariable logistic regression analysis. Gray solid dots indicate the distribution of each included patient. Solid blue lines are multivariable regression lines, with gray regions showing 95% confidence intervals. Adjustment factors are the same as those in Model 3 of Table 2. [file Image_4.TIF]

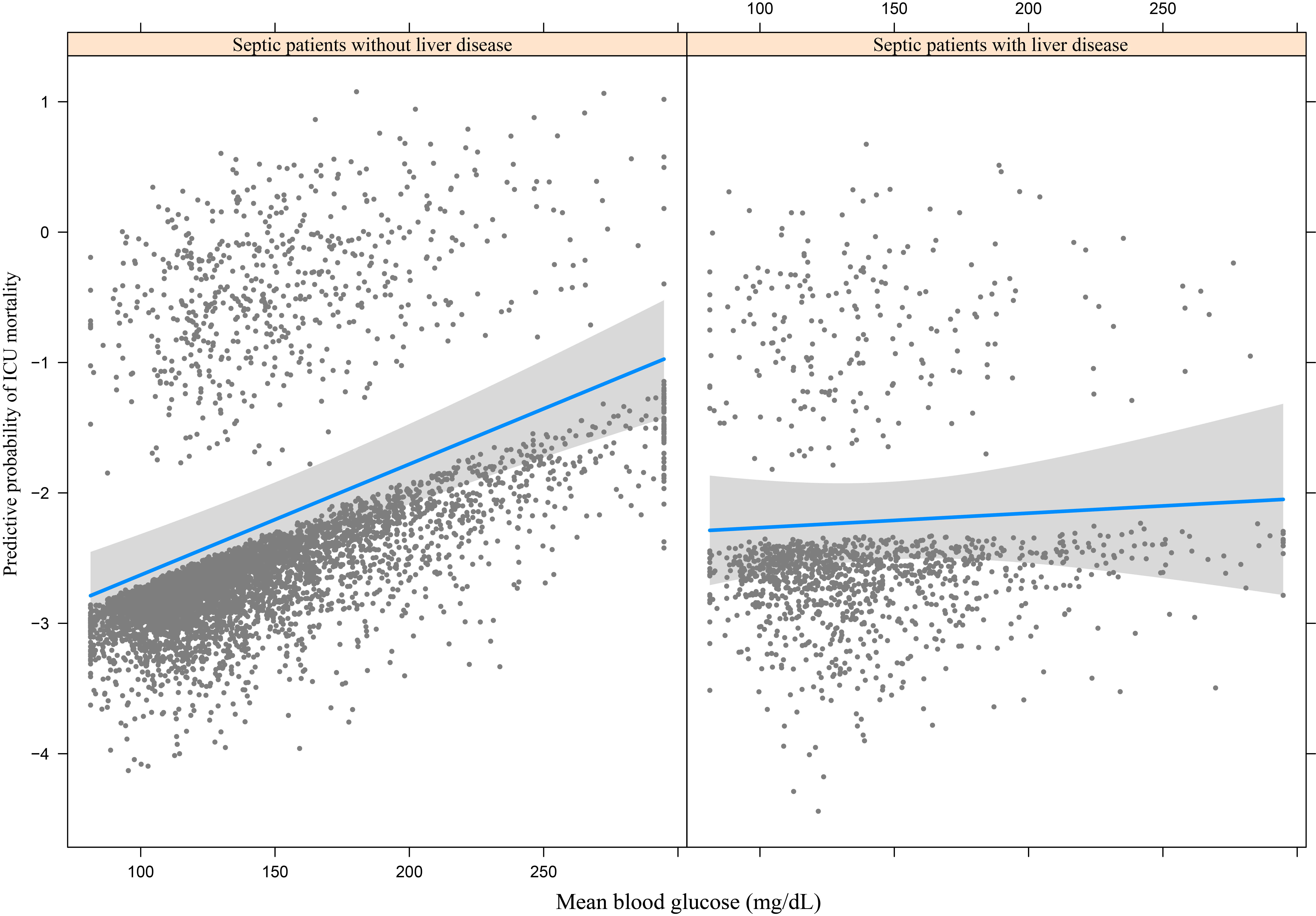

Supplement: Supplementary Figure 5 — The interaction between mean blood glucose (MBG) and liver disease. The abscissa and ordinate respectively represent the MBG values and predictive risk probability of ICU mortality by multivariable logistic regression analysis. Gray solid dots indicate the distribution of each included patient. Solid blue lines are multivariable regression lines, with gray regions showing 95% confidence intervals. Adjustment factors are the same as those in Model 3 of Table 2. [file Image_5.TIF]

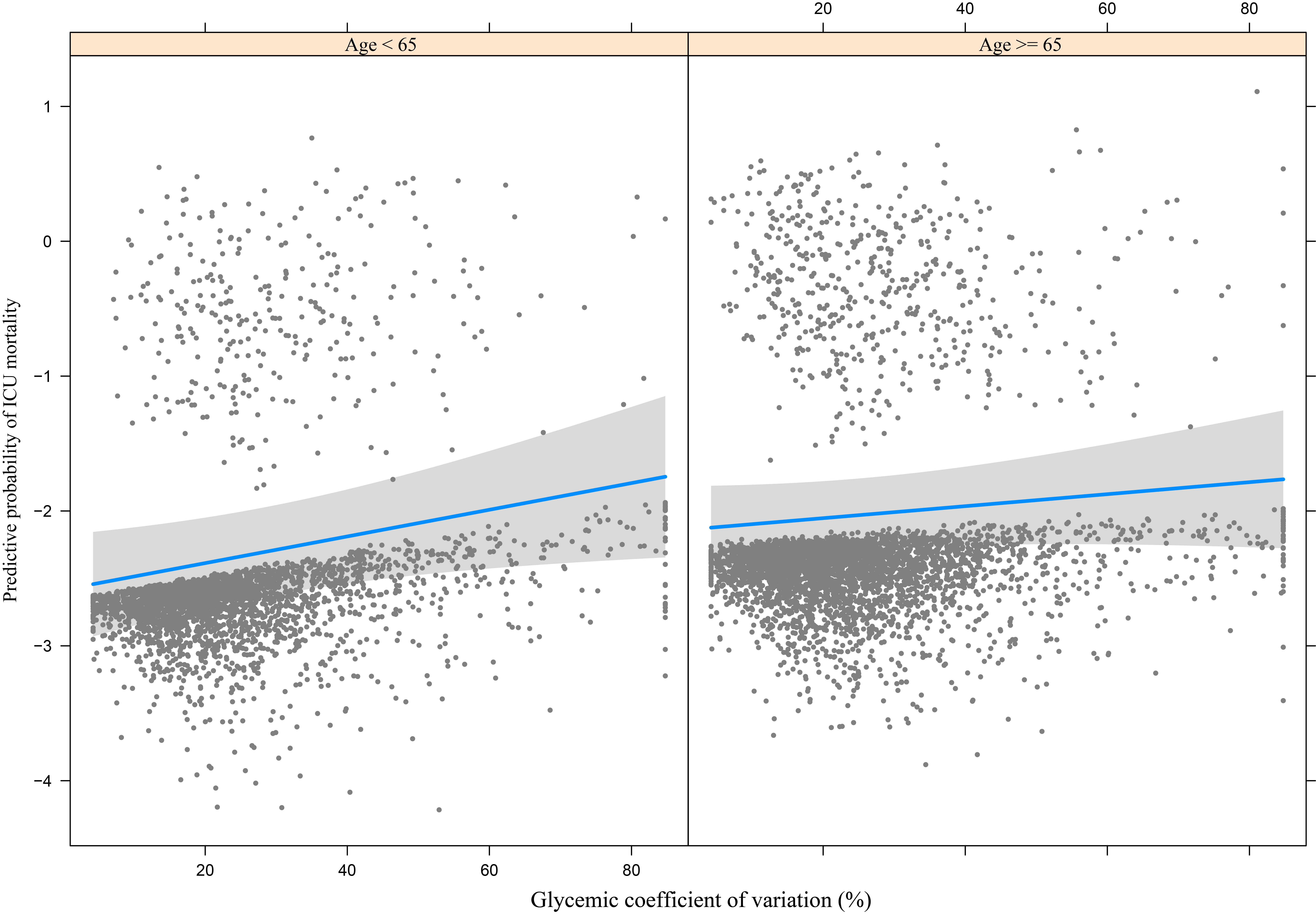

Supplement: Supplementary Figure 6 — The interaction between the glycemic coefficient of variation (GluCV) and age. The abscissa and ordinate, respectively, represent the GluCV values and predictive risk probability of ICU mortality by multivariable logistic regression analysis. Gray solid dots indicate the distribution of each included patient. Solid blue lines are multivariable regression lines, with gray regions showing 95% confidence intervals. Adjustment factors are the same as those in Model 3 of Table 2. [file Image_6.TIF]

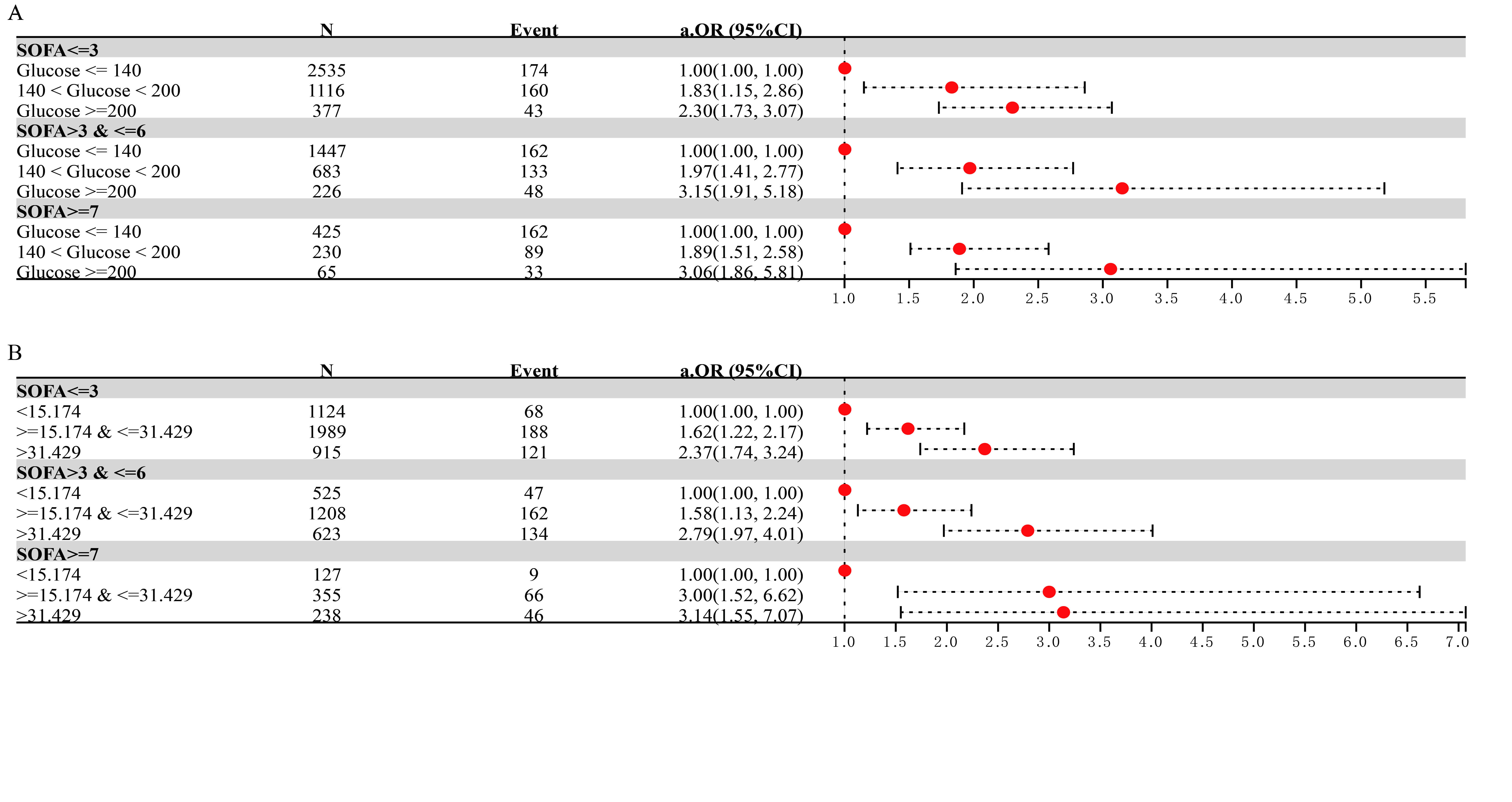

Supplement: Supplementary Figure 7 — The associations between MBG and GluCV with the ICU mortality of sepsis patients in different severity degrees according to the initial SOFA score. [file Image_7.TIF]

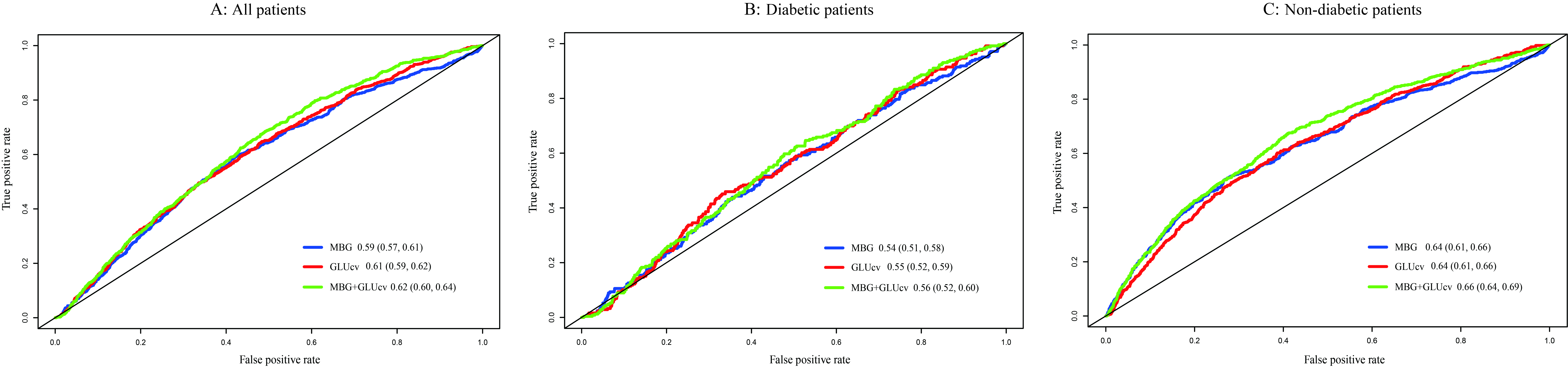

Supplement: Supplementary Figure 8 — The ROC curve of the mean blood glucose (MBG), glycemic coefficient of variation (GluCV), and MBG + GluCV in all septic patients (A), diabetic patients (B), and non-diabetic patients (C). [file Image_8.TIF]
