# Supplementary material for: Association of Blood Glucose Level and Glycemic Variability With Mortality in Sepsis Patients During ICU Hospitalization
Source: Front Public Health. 2022 Apr 29;10:857368. doi: 10.3389/fpubh.2022.857368 (PMC9099235; doi:10.3389/fpubh.2022.857368)
Supplement: Supplementary Table 1 — Comparison of performance of MBG, GluCV, and MBG combined with GluCV in predicting the ICU mortality of septic patients. [file Table_1.DOCX]

**Supplementary Table 1: Comparison of performance of MBG, Glu_CV_ and MBG combined with Glu_CV_ in predicting the ICU mortality of septic patients**

| **Population** | **Indicator** | **AUROC (95% CI)** | **SE** | **SP** | **P-value** |
| --- | --- | --- | --- | --- | --- |
| All septic patients | MBG | 0.59 (0.57, 0.61) | 0.60 | 0.57 | *Reference* |
|  | Glu_CV_ | 0.61 (0.59, 0.62) | 0.65 | 0.52 | *0.32* |
|  | MBG  +  Glu_CV_ | 0.62 (0.60, 0.64) | 0.69 | 0.51 | *< 0.001** |
| Diabetic patients | MBG | 0.54 (0.51, 0.58) | 0.52 | 0.57 | *Reference* |
|  | Glu_CV_ | 0.55 (0.52, 0.59) | 0.59 | 0.52 | *0.65* |
|  | MBG  +  Glu_CV_ | 0.56 (0.52, 0.60) | 0.60 | 0.52 | *0.13* |
| Non-diabetic patients | MBG | 0.64 (0.61, 0.66) | 0.50 | 0.73 | *Reference* |
|  | Glu_CV_ | 0.64 (0.61, 0.66) | 0.60 | 0.61 | *0.98* |
|  | MBG  +  Glu_CV_ | 0.66 (0.64, 0.69) | 0.67 | 0.60 | *< 0.001** |

The P-value was calculated by comparing the AUC of Glu_CV_ or MBG + Glu_CV_  with MBG using the DeLong test;

MBG, mean blood glucose; Glu_CV,_ glycemic coefficient of variation; AUROC, area under the receiver operating characteristic curve; SE, sensitivity; SP, specificity.
